# Dual Pharmacological Targeting of the MAP Kinase and PI3K/mTOR Pathway in Preclinical Models of Colorectal Cancer

**DOI:** 10.1371/journal.pone.0113037

**Published:** 2014-11-17

**Authors:** Todd M. Pitts, Timothy P. Newton, Erica L. Bradshaw-Pierce, Rebecca Addison, John J. Arcaroli, Peter J. Klauck, Stacey M. Bagby, Stephanie L. Hyatt, Alicia Purkey, John J. Tentler, Aik Choon Tan, Wells A. Messersmith, S. Gail Eckhardt, Stephen Leong

**Affiliations:** 1 Division of Medical Oncology, School of Medicine, University of Colorado, Anschutz Medical Campus, Aurora, Colorado, United States of America; 2 Department of Pharmaceutical Sciences, Skaggs School of Pharmacy and Pharmaceutical Sciences, University of Colorado, Anschutz Medical Campus, Aurora, Colorado, United States of America; 3 University of Colorado Cancer Center, University of Colorado Anschutz Medical Campus, Aurora, Colorado, United States of America; Université de Sherbrooke, Canada

## Abstract

**Background:**

The activation of the MAPK and PI3K/AKT/mTOR pathways is implicated in the majority of cancers. Activating mutations in both of these pathways has been described in colorectal cancer (CRC), thus indicating their potential as therapeutic targets. This study evaluated the combination of a PI3K/mTOR inhibitor (PF-04691502/PF-502) in combination with a MEK inhibitor (PD-0325901/PD-901) in CRC cell lines and patient-derived CRC tumor xenograft models (PDTX).

**Materials and Methods:**

The anti-proliferative effects of PF-502 and PD-901 were assessed as single agents and in combination against a panel of CRC cell lines with various molecular backgrounds. Synergy was evaluated using the Bliss Additivity method. In selected cell lines, we investigated the combination effects on downstream effectors by immunoblotting. The combination was then evaluated in several fully genetically annotated CRC PDTX models.

**Results:**

The *in vitro* experiments demonstrated a wide range of IC_50_ values for both agents against a cell line panel. The combination of PF-502 and PD-901 demonstrated synergistic anti-proliferative activity with Bliss values in the additive range. As expected, p-AKT and p-ERK were downregulated by PF-502 and PD-901, respectively. In PDTX models, following a 30-day exposure to PF-502, PD-901 or the combination, the combination demonstrated enhanced reduction in tumor growth as compared to either single agent regardless of KRAS or PI3K mutational status.

**Conclusions:**

The combination of a PI3K/mTOR and a MEK inhibitor demonstrated enhanced anti-proliferative effects against CRC cell lines and PDTX models.

## Introduction

Two of the most implicated cellular pathways in cancers are the phosphatidylinositol-3 kinases (PI3K) and the mitogen activated protein kinase (MAPK) pathways. The class I (PI3K) are heterodimeric lipid kinases that comprise a regulatory p85 subunit and a catalytic p110 subunit [1]. PI3K phosphorylates the 3-hydroxyl group of phosphatidylinositol, participating in a variety of signaling pathways important for cancer such as proliferation, differentiation, chemotaxis, survival, trafficking, and glucose homeostasis [2], [3]. Because of its diverse cellular function, the PI3K axis is highly implicated in human cancers; up to 30% of all human cancers have a mutation in a PI3K pathway component [4]. In colorectal cancer (CRC), the *PIK3CA* gene, encoding the p110α catalytic subunit of class I PI3Ks, has been found to be mutated in 10–20% of CRC tumor specimens [5].

A downstream component of the PI3K signaling pathway is the mammalian target of rapamycin (mTOR). Cell growth is one of the primary functions governed by mTOR; activation of mTOR via the PI3K/AKT pathway is critical for the cell in balance nutrient uptake and growth, and aberrant hyperactivation of this pathway contributes to tumorigenesis [6], [7]. The role of mTOR in these cellular functions makes it an attractive target for inhibition; since the development of rapamycin forty years ago, many first and second generation mTOR inhibitors have been synthesized and are in various stages of clinical and preclinical development [8], [9].

The MAPK/ERK (MEK) complexes are components of the Ras/Raf signaling axis. Signaling through this pathway results in increased proliferation and resistance to apoptosis, whereas constitutive activation contributes to chemoresistance in several cancers [10], [11]. Mutations in KRAS, NRAS, or BRAF (all upstream of the MEK complexes) are very common in CRC, and have been found in 50–60% of tumor samples [12], [13]. A variety of agents have been developed that target EGFR, RAS, RAF, or MEK, many of which are in clinical trials and some of which are already approved [14].

Crosstalk between the PI3K/AKT/mTOR pathway exists: for example, PI3K can be activated by RAS, and the tumor suppressor tuberin (a negative regulator of mTOR) is a direct substrate of ERK [15]–[17]. It has been found also that co-occurrence of alterations in the PI3K-AKT-mTOR and RAS-RAF-MEK pathways occurs in one third of CRC samples, suggesting that simultaneous inhibition of both pathways may be necessary for therapeutic benefit [12]. Additionally, it is thought that the RAS-RAF-MEK signaling axis may act as a compensatory mechanism with inhibition of the PI3K-AKT-mTOR pathway, and vice versa [18], [19]. The evidence of extensive cross-talk between these pathways has created great interest in simultaneous inhibition, with several different strategies now in development [20].

To explore the efficacy of simultaneous inhibition of both the PI3K-AKT-mTOR and the RAS-RAF-MEK pathways, we examined the combination of PF-04691502 (PF-502) with PD-0325901 (PD-901). PF-502 is an orally bioavailable, potent ATP-competitive kinase inhibitor of both class I PI3Ks and mTOR [21], [22]. In a recently completed a Phase I clinical trial, PF-502 was found to be well tolerated with fatigue, decrease appetite, nausea hyperglycemia, rash, vomiting, diarrhea and mucosal inflammation being the most commonly seen adverse events. However majority of these were Grade 1 or 2. [23] PD-901 is a highly potent, oral, small-molecule inhibitor of MEK1 and MEK2 that demonstrated some activity in early clinical trials and was associated with toxicities characteristic of this class of agents: rash_,_ diarrhea, fatigue, nausea and visual disturbance, [24]. PD-901 is currently in Phase I clinical trials for the treatment of CRC in combination with PKI-587 (PF-0521384), a dual PI3K/mTOR inhibitor with similar potency to PF-502 [24]–[26]. One benefit of this combination is that through inhibition of the upstream PI3K, feedback upregulation of AKT upon mTOR inhibition is avoided [27].

In the current study we describe the enhanced anti-tumor activity of the dual PI3K/mTOR inhibitor PF-502 and the MEK inhibitor PD-901 in *in vitro* and *in vivo* models of colorectal cancer.

## Materials and Methods

### Chemicals and Reagents

PF-04691502 (PF-502) and PD-0325901 (PD-901) were provided by Pfizer Inc. (San Diego, CA). For *in vitro* work, both agents were dissolved in 100% DMSO at a concentration of 10 mM. For *in vivo* studies, PF-502 was suspended in 0.5% methylcellulose in water, and PD-901 was suspended in 0.5% hydroxypropyl methylcellulose, 0.2% tween-80 in water.

### Cell Lines and Culture

Human colorectal cancer cell lines were obtained from American Type Culture Collection, DSMZ, or the Korean Cell Line Bank. Geo cells, described previously [28], were kindly provided by Dr. Fortunato Ciardiello (Cattedra di Oncologia Medica, Dipartimento Medico-Chirurgico di Internistica Clinica e Sperimentale “F Magrassi e A Lanzara,” Seconda Universita’ degli Studi di Napoli, Naples, Italy). KM12L4, KM12C, and KM20, described previously [29], were all kindly provided by Scott Kopetz (MD Anderson Cancer Center, Houston, TX). All cells were routinely cultured in RPMI 1640. All medium was supplemented with 10% FBS, 1% penicillin–streptomycin, and 1% MEM nonessential amino acids. All cells were kept at 37°C under an atmosphere containing 5% CO_2_. Cells were routinely tested for the presence of mycoplasma (MycoAlert; Cambrex BioScience). All CRC cell lines used in this study have been fully characterized and authenticated in the University of Colorado Cancer Center DNA Sequencing and Analysis Core.

### Sulforhodamine B (SRB) and CyQuant Cell Proliferation Assays

Cells were seeded in 96-well clear plates at 2000–8000 cells per well, depending on cell line properties. For the CyQuant assay, cells were plated in black-walled plates. After 24 h, cells were incubated for 72 h with media only or varying concentrations of PD-901 (0.015 umol/L-1 umol/L) or PF-502 (0.08 umol/L-5 umol/L), as single agents or in combination. For the CyQuant assay, plates were prepared and read according to the manufacturer’s protocol (Life Technologies, Carlsbad, CA). Sulforhodamine B assay (SRB) was performed as described previously [30]. Briefly, cells were fixed with 10% tricholoracetic acidat 4°C for 30 minutes, washed 3X with water, and dried at RT. They were then stained with a sulforhodamine B (SRB) solution (0.4% w/v SRB in 1% acetic acid) for 20 min at room temperature washed 3× with 1% acetic acid, and remaining SRB was solubilized with 10 mM unbuffered Tris. The absorbance was read at 492 nm, using the Synergy 2 plate reader (Biotek, Winooski, VT). Data was normalized to absorbance of no drug (ND). A combination effect was analyzed using the Bliss Additivity model as described previously [31].

### Caspase 3/7 Activity

The Caspase-Glo 3/7 Luminescent assay (Promega, Milwaukee, WI) was used as an indicator of apoptotic activity through caspase 3 and 7 activity. Cells were seeded in white-walled plates at 2000–8000 cells per well, depending on cell line properties. After 24 h, cells were incubated for the indicated time point with media only or varying concentrations of PD-901 or PF-502, as single agents or in combination. Data is presented as fold-change, normalized to control.

### Clonogenic Assays

Cells were seeded at 2000 cells per well of a 6-well plate and allowed to attach overnight. PD-901 or PF-502 was added as single agents or in combination and incubated for 72 hours. After 72 hours cells were washed in complete media and media without drug was added back for an additional 72 hours. Cells were fixed with 100% methanol for 30 min and stained with 2% crystal violet. The crystal violet was then solubilized in 1% acetic acid and the supernatant was transferred to a 96-well plate. The absorbance was read at 565 nm, using the Synergy 2 plate reader (Biotek, Winooski, VT). The data was normalized to control.

### Immunoblotting and Antibody Array

Cells were seeded in 6-well plates and allowed to attach for 24 h. Cells were then incubated for 24 h with either PD-901 or PF-502, or the combination. Cells were then washed with PBS and lysed with RIPA buffer (Cell Signaling, Danvers, MA). After sonication and centrifugation, a total of 30 ug of protein lysate was loaded onto a NuPage gel (Life Technologies, Carlsbad, CA), electrophoresed, and transferred to a nitrocellulose membrane using the iBlot (Invitrogen, Carlsbad, CA). The membrane was blocked and probed overnight with primary antibodies, washed for 10 minutes 3× with, and probed with DyLight secondary antibodies (Cell signaling, Danvers, MA), and imaged using the Licor Odyssey (Licor, Lincoln, NE). All primary antibodies were purchased from Cell Signaling Technology (Danvers, MA) and diluted as per the manufactures’ instructions. For the antibody array, excised tumors were lysed in RIPA buffer using a FastPrep24 tissue homogenizer (MP Biologicals, Santa Ana, CA). Protein concentration was normalized and added to the PathScan Stress and Apoptosis Signaling Antibody Array as per the manufactures’ instructions. Array was developed using the Licor Odyssey (Licor, Lincoln, NE). Intensity of each spot was analyzed using the Image Studio software (Licor, Lincoln, NE) and the density of each spot was graphed.

### Patient-derived Xenograft Studies

Five to six week-old female athymic nude mice (Harlan Labs, Indianapolis, IN) were used for all animal studies, caged in groups of 5, kept on a 12 hour light/dark cycle, and given sterile food and water *ad libitum*. The patient-derived xenografts were generated as previously described [28]. Briefly, a tumor specimen was collected at the time of surgery from a consenting patient at the University of Colorado Hospital. Tumor material remaining after histopathologic analysis was cut into 2 to 3 mm^3^ pieces, submerged in Matrigel, and implanted subcutaneously into the flank of five nude mice. After tumors were expanded through at least the F3 generation, they were excised, cut, and injected into the left and right flanks of approximately 25 mice for each xenograft study (50 total tumors divided between four groups: Vehicle Control, PF-502 single agent, PD-901 single agent, and PF-502/PD-901 combination). When the average tumor size reached a volume of approximately 200 mm^3^, mice were randomized into the four different groups. Mice were monitored daily for signs of toxicity and weighed twice weekly. Treatment was administered once daily (10 mg/kg PF-502 and/or 1.0 mg/kg PD-901) by oral gavage. Tumor volume (equation for volume = (length×width^2^)×0.52) was evaluated twice per week with digital calipers, using the Study Director software package (Studylog Systems, South San Francisco, CA). Tumor growth rates were determined for each individual tumor by fitting tumor volume data over the course of the treatment period to an exponential growth rate equation with SAAM II v. 2.3. (The Epsilon Group, Charlottesville, VA). At the end of treatment, mice were sacrificed by CO_2_ overdose followed by cervical dislocation prior to removal of the tumors for further analysis.

### Data Analysis

One-way ANOVA analyses with a Tukey post-test was used to determine statistical significance between multiple groups. Analyses were performed with Prism version 5.0, *p-*values <0.05 were considered statistically significant.

### Ethics Statement

All animal work and care were performed under the guidelines of the Institutional Animal Care and Use Committee (IACUC). Specific approval for the mouse experiments was obtained with the protocol 51410(08)1E entitled “Development and Maintenance of Primary GI Tumor Bank for Designing Rational Treatment using Mouse Tumor Explants”. All reasonable efforts were made to ameliorate suffering, including anesthesia for painful procedures. The human tumors were obtained using Colorado Multiple Institutional Review Board (COMIRB) approved protocol number 08–0439. Consent for tumor retrieval was written.

## Results

### The effects of PF-502 and PD-901 as single agents on proliferation of colorectal cancer cell lines

**Figure 1:**
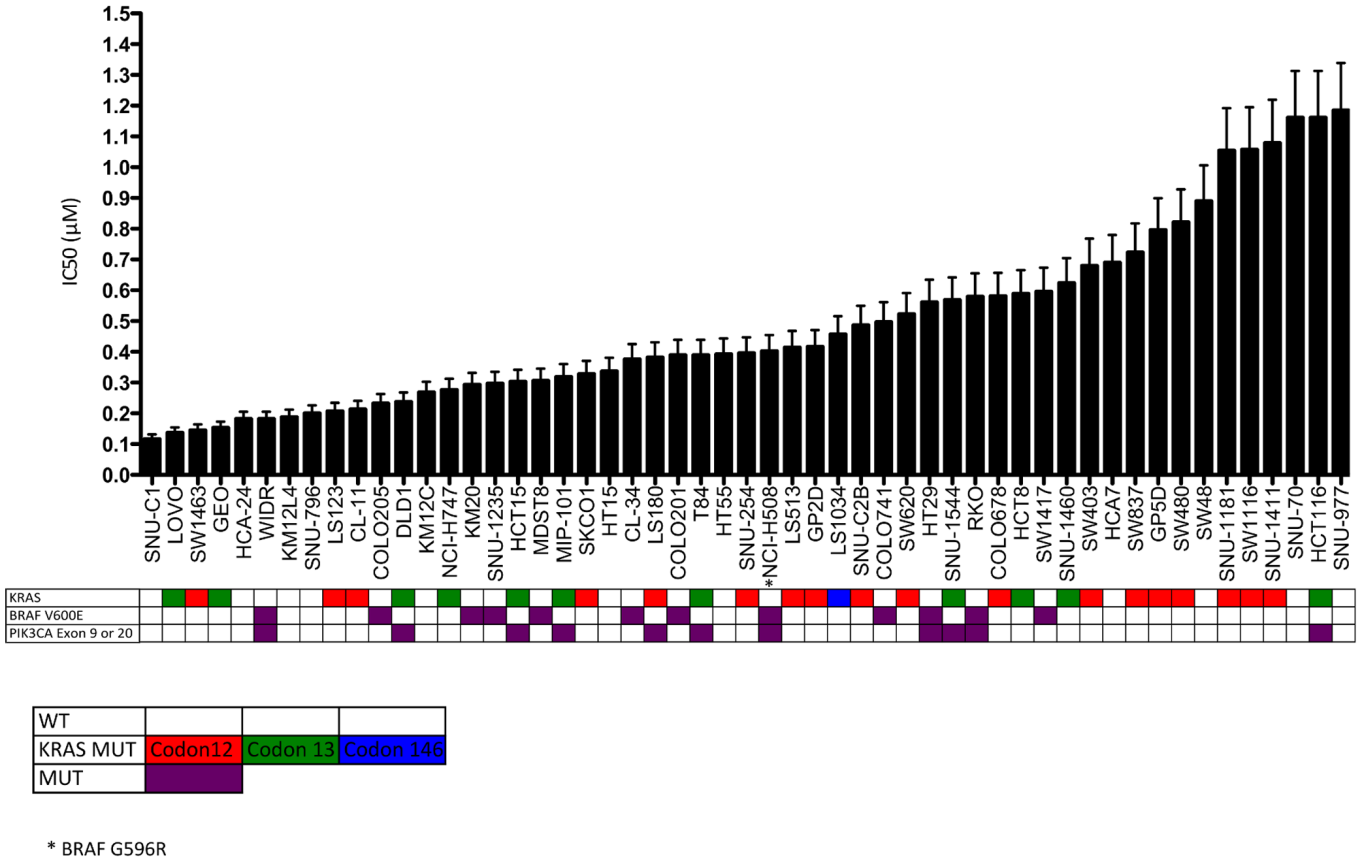
Proliferative effects on colorectal cancer cell lines plotted as IC_50_ following exposure to the PI3K/mTORi, PF-04691502 (0–5 umol/L). Cells were plated in 96 well plates and allowed to adhere for 24 hours. Cells were exposed to increasing concentrations of compound for 72 hours and proliferation was assessed using CyQuant assay. Proliferaion assays were conducted in triplicate and IC_50_ values were calculated from the average proliferation. Mutational status of KRAS, BRAF, and PIK3CA are in colored boxes.

CRC cell lines were exposed to increasing concentrations of PF-502 and PD-901 for 72 hours, proliferation was assessed and IC_50_ values were calculated from average proliferation of triplicate experiments. As depicted in **Figures 1** **and** **2** there was a greater than 10-fold difference in IC_50_s between sensitive and resistant CRC cell lines that did not correlate with mutational status of KRAS, BRAF, or PIK3CA. Four CRC cell lines with varying genotypes were chosen for further analysis.

**Figure 2:**
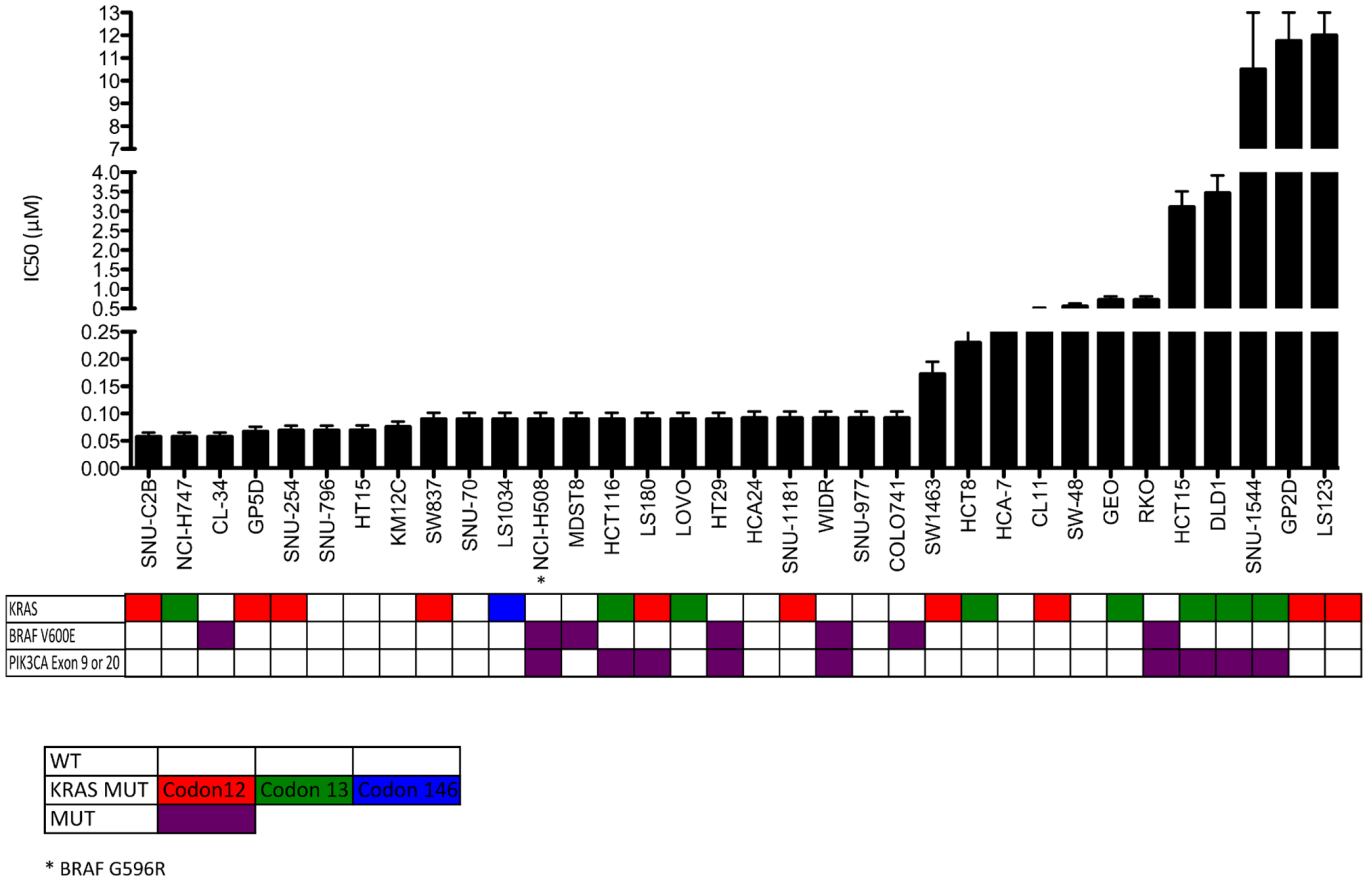
Proliferative effects on colorectal cancer cell lines plotted as IC_50_ following exposure to the MEKi, PD-0325901 (0–1 umol/L). Cells were plated in 96 well plates and allowed to adhere for 24 hours. Cells were exposed to increasing concentrations of compound for 72 hours and proliferation was assessed using SRB assay. Proliferation assays were conducted in triplicate and IC_50_ values were calculated from the average proliferation. Mutational status of KRAS, BRAF, and PIK3CA are in colored boxes.

### Combinatorial effects of the PI3K/mTORi PF-502 and the MEKi, PD-901 in colorectal cancer cell lines

LOVO (KRAS^G13D^/PIK3CA ^WT^), HCT116 (KRAS^G13D^/PIK3CA^H1047R^), WIDR (BRAF^V600E^/PIK3CA^P449T^), and Geo (KRAS^G13D^/PIK3CA ^WT^) were chosen for further combination analysis.

**Figure 3:**
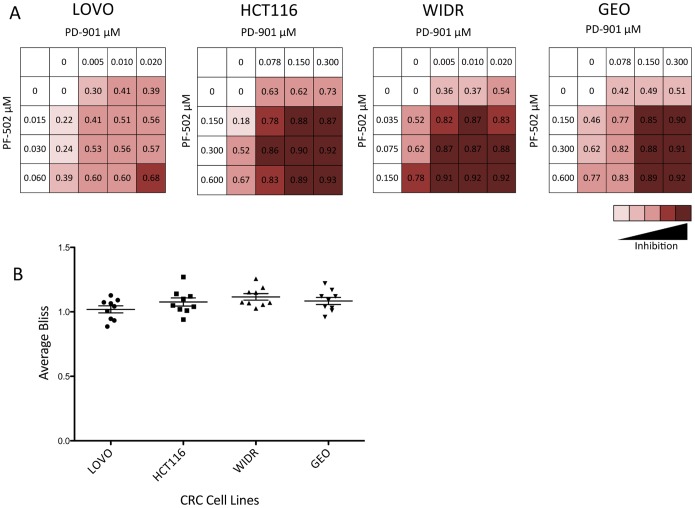
Growth inhibition of the PI3K/mTORi, PF-04691502 (PF-502) combined with the MEKi, PD-0325901 (PD-901) on four colorectal cancer cell lines. LOVO (KRAS^G13D^), HCT116 (KRAS^G13D^/PIK3CA^H1047R^), WIDR (BRAF^V600E^/PIK3CA^P449T^), GEO (KRAS^G13D^). Cells were exposed to various combinations of PF-502 and PD-901 for 72 hours. (A) Fraction inhibited was graphed following exposure to single agent and combination. (B) Bliss additivity was calculated and to assess combinatorial effects. Average bliss scores were graphed.

**Figure 4:**
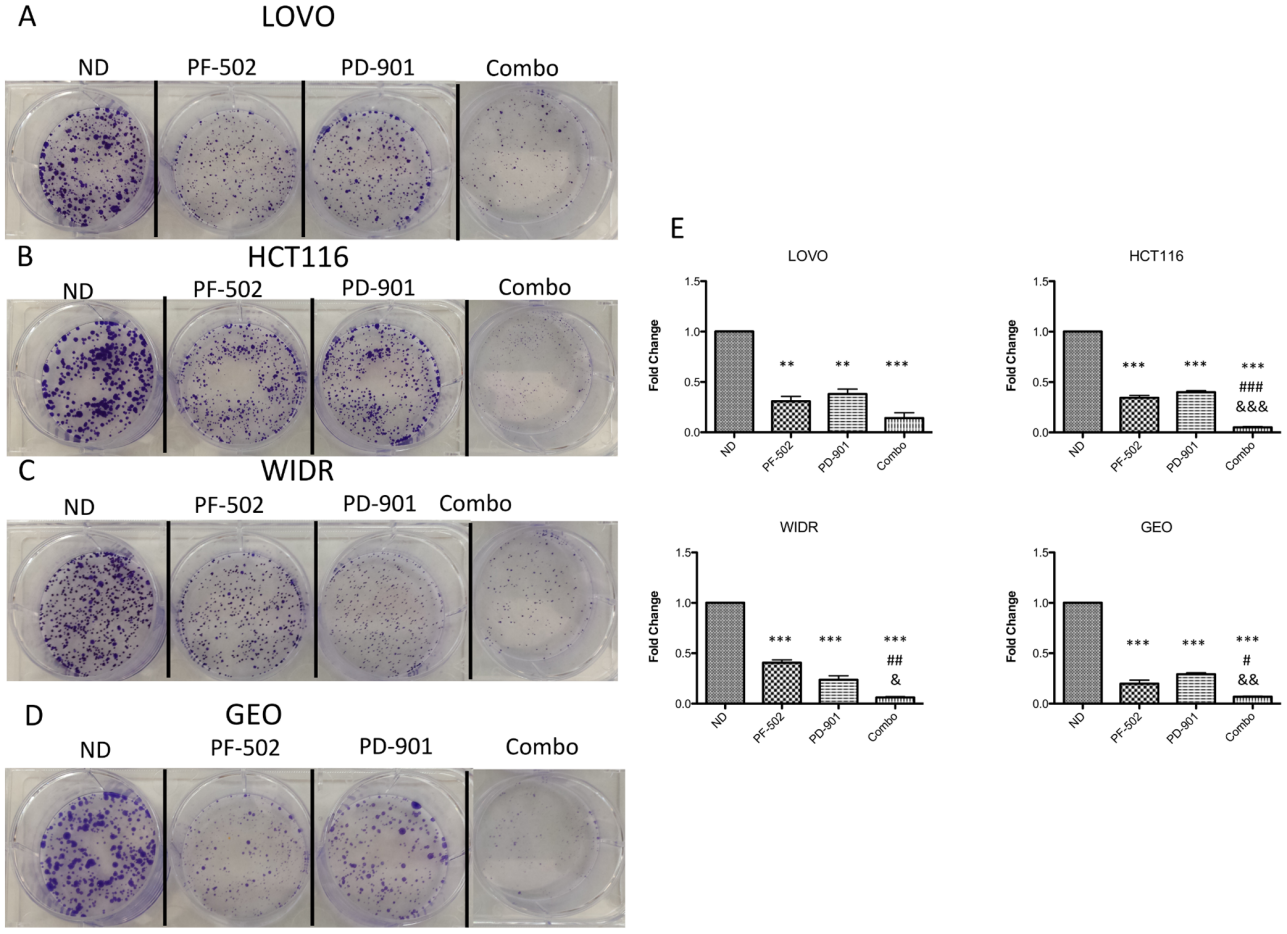
Clonogenic analysis with the combination of the PI3K/mTORi, PF-04691502 (PF-502) combined with the MEKi, PD-0325901 (PD-901) on four colorectal cancer cell lines. (A) LOVO, (B) HCT116, (C) WIDR, and (D) GEO were plated in 6 well plates and exposed to single agent the PI3K/mTORi, PF-04691502, the MEKi, PD-0325901 or the combination for 72 hours. Drug was removed and replaced with media to allow for regrowth of clones. Cells were then stained with crystal violet and photographed. (E) The crystal violet was solubilized in 1% acetic acid and the absorbance was assessed on a plate reader. The data was normalized to control and graphed. All data presented as mean±SD, ANOVA Tukey’s adjusted p values: *p,0.05, **p<0.01, ***p<0.001 vs vehicle, #*p*,0.05, ##*p*<0.01, ###*p*<0.001 vs PF-502, &*p*,0.05, &&*p*<0.01, &&&*p*<0.001 vs PD-901. All data represents three independent experiments.

Each of the 4 CRC cell lines were exposed to varying concentrations of the PI3K/mTORi, PF-502 and the MEKi PD-901 as single agents or in combination for 72 hours. Proliferation was assessed using the CyQuant assay and inhibition values are presented in **Figure 3A**. The combination demonstrated a greater effect on inhibition of proliferation in all four CRC cell lines tested at various concentrations. This data was confirmed in the CRC cell lines using the Bliss Additivity model. Bliss values were calculated as described in the Materials and Methods and the score for each combination is presented in **Figure 3B**. HCT116, WIDR and GEO both show average Bliss scores slightly above 1 demonstrating a greater than additive effect (1.08±0.09, 1.11±0.08, 1.08±0.08, respectively) while LOVO demonstrated roughly an additive effect (1.019±0.08,). To determine if the combination effect observed was associated with irreversible inhibition of proliferation, a clonogenic assay was performed. The four CRC cell lines were exposed to 0.6 µmol/L of PF-502 and 0.3 µmol/L of PD-901 as single agents and in combination. All four cell lines exhibited a reduction in clonogenicity of the combination versus either single agent whereas the HCT116, GEO, and WIDR demonstrated statistically significant differences. (**Figure 4**).

**Figure 5:**
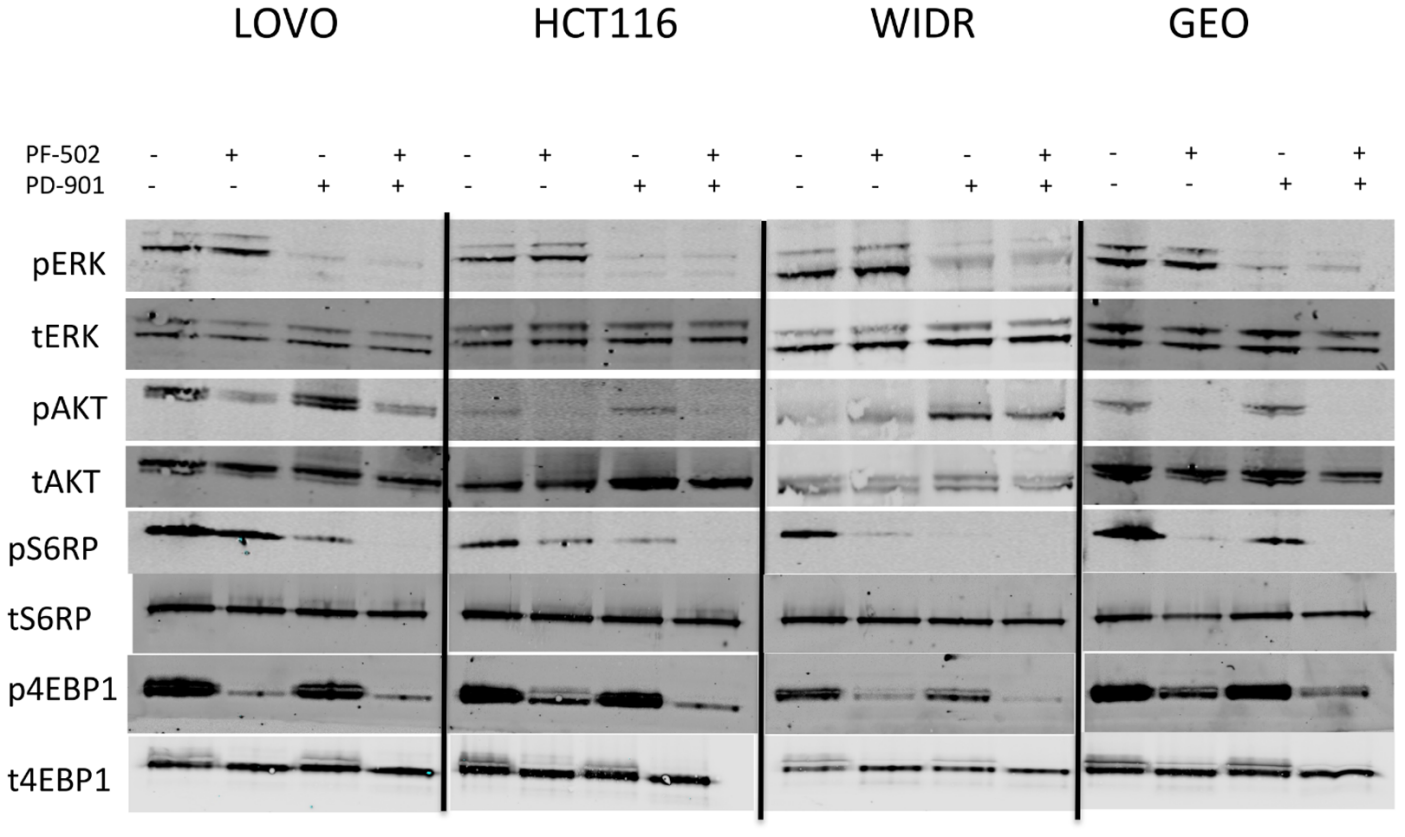
Effect of single agent the PI3K/mTORi, PF-04691502, the MEKi, PD-0325901 or the combination on downstream effector proteins assessed by immunoblotting.

We also characterized the effects of the PI3K/mTORi, PF-502 and the MEKi, PD-901 on established downstream effectors of relevant pathways. PF-502 has been shown to decrease AKT, 4EBP1 and S6RP phosphorylation [22], whereas PD-901 inhibits phosphorylation of ERK [32], [33]. To validate the effects of these 2 agents and look for evidence of a cooperative effect, modulation of downstream targets in the MAPK and PI3K pathways were analyzed. As depicted in **Figure 5**, treatment with PF-502 or PD-901 induced decreases in pAKT, pS6RP, P4EBP1, and pERK respectively. Interestingly, upon exposure to the MEKi, PD-901 we observed a more pronounced decrease in pS6RP than what was observed in the PF-502 treated cells in three out of the four CRC cell lines. These decreases were largely maintained in the combination.

### Apoptosis is enhanced following exposure to the combination of PF-502 and PD-901 in colorectal cancer cell lines

**Figure 6:**
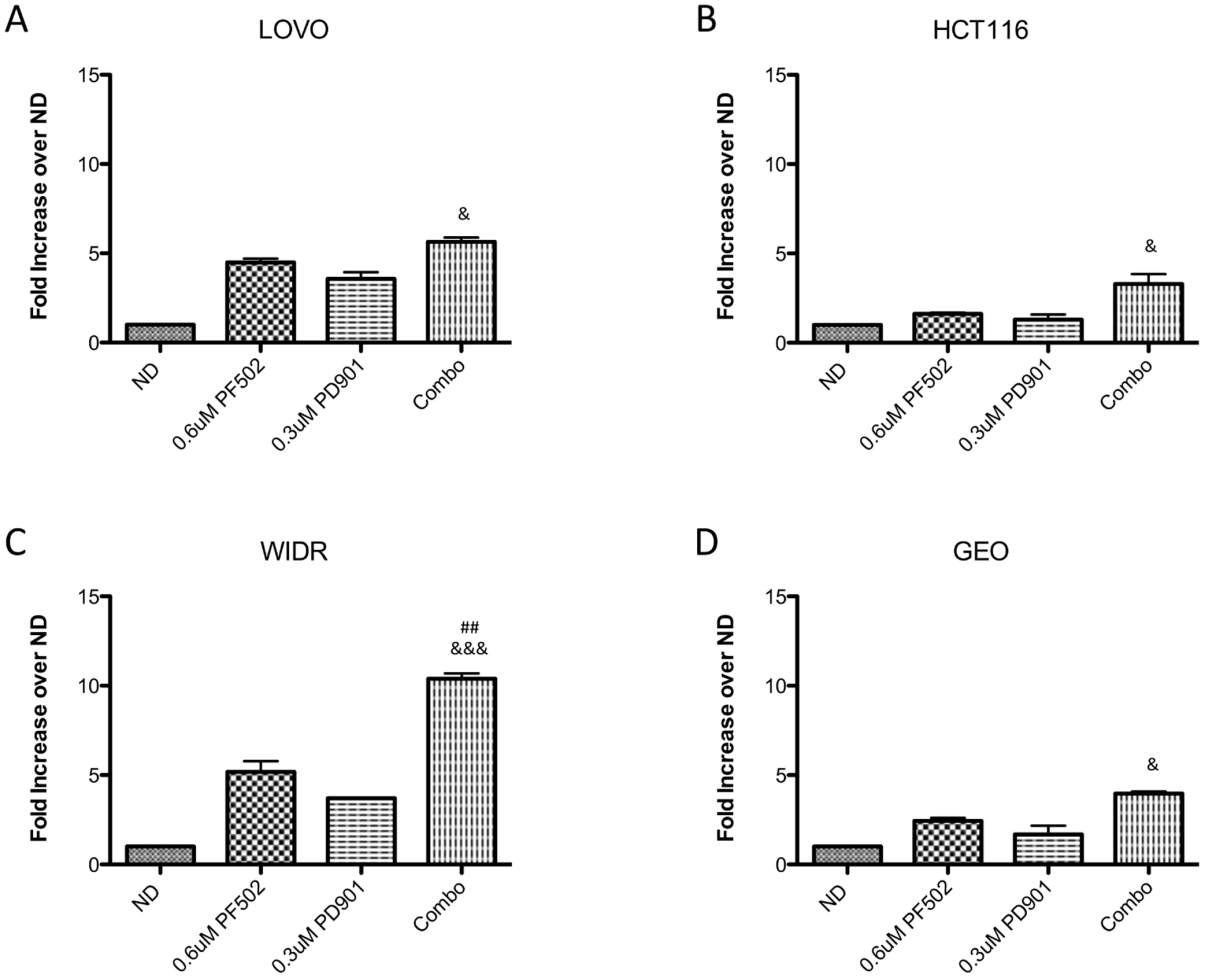
Effect of single agent the PI3K/mTORi, PF-04691502, the MEKi, PD-0325901 or the combination on Caspase 3/7 activity. All data presented as mean±SD, ANOVA Tukey’s adjusted p values: *p,0.05, **p<0.01, ***p<0.001 vs vehicle, #*p*,0.05, ##*p*<0.01, ###*p*<0.001 vs PF-502, &*p*,0.05, &&*p*<0.01, &&&*p*<0.001 vs PD-901. All data represents three independent experiments.

The clonogenic data indicated that the combination resulted in sustained antiproliferative effects, thus we measured apoptosis as another phenotypic endpoint. To do this we exposed the four CRC cell lines to 0.6 µmol/L of PF-502 and 0.3 µmol/L of PD-901 as single agents and in combination for 24 hours. Apoptosis was measured by the Caspase Glo 3/7 assay. As depicted in **Figure 6**, although there was a trend towards increased apoptosis with the combination in all cell lines, in WIDR cells these effects were statistically significant compared to both single agents.

### Antitumor effects of PF-502 and PD-901 in patient derived tumor xenografts

**Figure 7:**
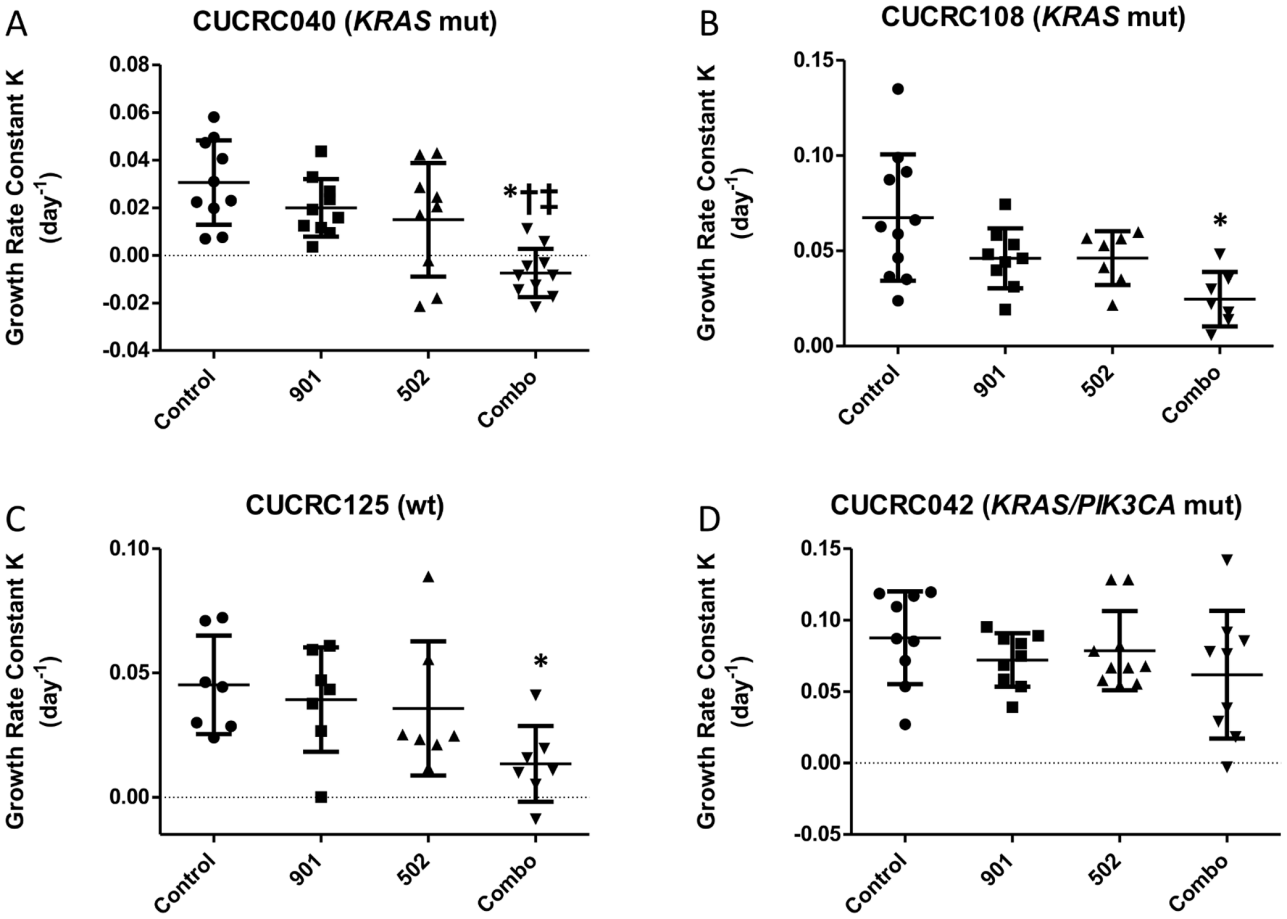
Tumor growth rate analysis on patient-derived tumor xenograft models (PDTX). Tumor growth rates were determined for each individual tumor by fitting tumor volume data over the course of the treatment period to an exponential growth rate equation. Each point represents a single tumor. Mean tumor growth rate ± standard deviation are represented by the bar and handles. All data presented as mean±SD, ANOVA Tukey’s adjusted p values: *P<0.05 vs. Control; ^†^P<0.05 vs. 901; ^‡^P<0.05 vs. 502.

Next to confirm the anti-tumor effects observed in our *in vitro* model, we exposed four patient derived tumor xenografts (PDTX) models with varying mutational status to the PI3K/mTORi, PF-502, the MEKi, PD901, or the combination. The anti-tumor effects of each compound was assessed by determining the growth rate of each individual tumor in each treatment group. As depicted in **Figure** **7A** **,** only the CUCRC040 model (*KRAS* ^G12V^/PIK3CA ^E542G^) demonstrated a significant benefit of the combination treatment over single agent treatments (p<0.05). The combination treatment was also statistically significant in the CUCRC108 (KRAS ^G12C^) and CUCRC125 (KRAS ^WT^) PDTX models compared to controls, but not compared to single agent treatment groups (**Figure 7B–C** **)**. And, the CUCRC042 (KRAS ^G13D^) model was relatively unresponsive to all treatments (**Figure 7D**). Tumor growth curves are shown in **Figure S1.**

### The combination of PF-502 and PD-901 inhibits pro-survival pathways and impacts apoptotic markers in PDTX models

**Figure 8:**
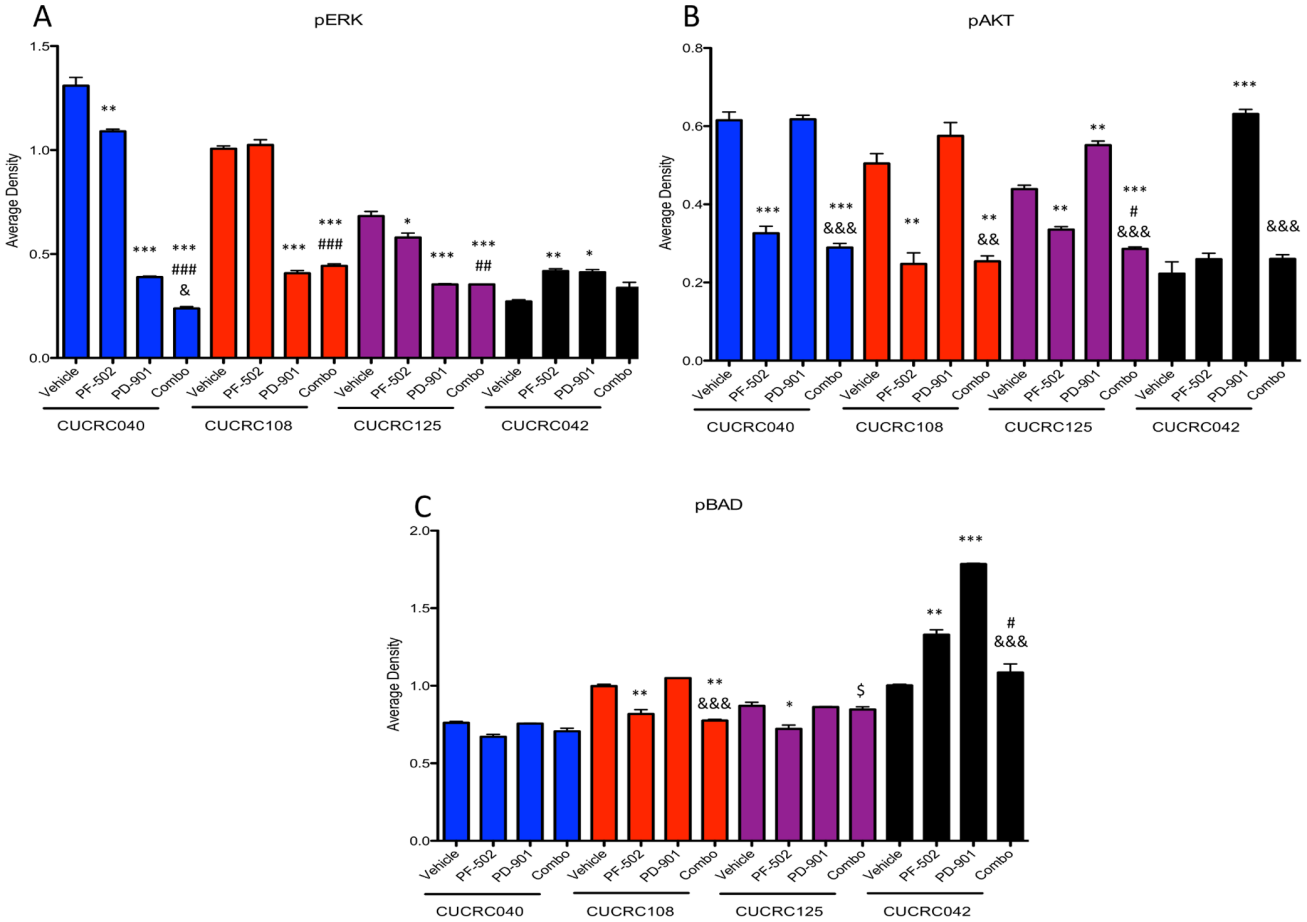
Effect of single agent the PI3K/mTORi, PF-04691502, the MEKi, PD-0325901 or the combination on downstream effector proteins assessed by antibody array. Total protein was purified from PDTX at the end of treatment and assessed on a stress and apoptosis antibody array. Density of the spots were obtained, and graphed. All data presented as mean±SD, ANOVA Tukey’s adjusted p values: *p,0.05, **p<0.01, ***p<0.001 vs vehicle, #*p*,0.05, ##*p*<0.01, ###*p*<0.001 vs PF-502, &*p*,0.05, &&*p*<0.01, &&&*p*<0.001 vs PD-901. All data is a representative of three separate tumors from each group.

To assess the effects on signaling pathways of the PI3K/mTORi, PF-502 and the MEKi, PD-901 in the PDTX models, an antibody array was used with the lysates of tumors collected from group for each model at the end of study. Tumors were lysed in RIPA buffer and applied to the PathScan Stress and Apoptosis Signaling Antibody Array at equal concentrations. The arrays were visualized on a Licor Odyssey and density/intensity of duplicate spots from three different tumors were normalized to the vehicle control and graphed. Levels of pERK and pAKT were decreased in the PD-901 and PF-502 treatment groups, compared to control, in the CUCRC040, CUCRC108 and CUCRC125 PDTX models, which is consistent with the tumor growth profiles and response observed in the these treatment groups (**Figure 8A–B** **and Figure S2**). Additionally, the downregulation of pERK and pAKT was maintained in the combination groups in these models. However, only in the CUCRC040 model was the level of pERK in the combination group actually lower than both single agent arms. The CUCRC042 resistant model did not show any decrease in pERK or pAKT (**Figure 8A–B** **and Figure S2**). Interestingly, in CUCRC042 treatment with PD-901 resulted in an increase in pAKT and an increase in pBAD (**Figure 8B–C** **and Figure S2**).

## Discussion

Approximately 40% of colorectal cancers harbors a mutation in the KRAS gene causing this pathway to be constitutively active. Other mutations in this pathway, including RAS (NRAS and HRAS) and BRAF comprise another 1–3% and 5–15%, respectively [34], [35]. PIK3CA mutations occur in approximately 15% of colorectal cancers and of these 8–9% have mutations in both PIK3CA and KRAS [34], [36]. With both of these major pro-survival/proliferative pathways active in colorectal cancers, in addition to the cross-talk/feedback that occurs between these pathways, there is a rationale to target both pathways with small molecule inhibitors.

Inhibitors of the RAS/RAF/MEK pathway have been around for over a decade and single agent clinical studies have been largely disappointing. For example, a Phase II trial with CI-1040, a first-generation MEK inhibitor, in advanced colorectal cancer did not demonstrate significant antitumor activity. However, a more recent Phase II trial with the second generation MEK inhibitor selumetinib demonstrated similar activity to the chemotherapeutic agent capecitabine in CRC patients [37], [38]. With modest results to single agent MEK inhibition in colorectal cancers, it seems obvious that combination therapy should be investigated.

Numerous preclinical studies have demonstrated that targeting both the RAS/RAF/MEK and the PI3K/AKT/mTOR pathway is more beneficial in numerous tumor types [39]–[42]. In ovarian cancer co-targeting of the PI3K/mTOR and RAS/MEK pathway demonstrated a synergistic inhibition of proliferation and induction of cell death [42]. Similarly, in basal-like breast cancer models, an enhanced inhibition of proliferation and an increase in apoptosis was observed *in vitro* and an increase in tumor growth inhibition was observed *in vivo* [39]. Most of the data in colorectal cancer has been in KRAS mutant models since this population is in need of new novel therapies. Roper *et. al*. demonstrated that combination PI3K and MEK inhibition promotes apoptosis and tumor regression in mouse models of colorectal cancers [41]. Another study in human colorectal PDTX models, demonstrated more of a tumor stasis effect, with and little regression suggesting this combination regimen may not translate into durable therapeutic effects [40].

In the current study we utilized both colorectal cancer cell lines and PDTX models to demonstrate that PI3K/MEK combination therapy is more active than single agent treatment in models of CRC with distinct molecular backgrounds. Both PF-502 and PD901 exhibited single agent activity against in a large panel of colorectal cancer cell lines irrespective of RAS or PIK3CA mutational status. When the combination was tested in KRAS mutant, BRAF mutant or KRAS/PIK3CA double mutants there was an enhanced combination effect at various concentrations with HCT116, WIDR and GEO exhibiting a slightly more robust combination effect when compared to LOVO. The ability to form colonies following exposure to the combination was also impaired indicating that inhibition of these pathways leads to a cytotoxic rather than cytostatic phenotype. This was further supported by an increase in apoptosis in the combination. It is interesting to note that upon treatment with the MEKi, PD901, a marked decrease was observed in pS6RP in three of the four CRC cell line models. This may be due to the fact that TORC1 activity, which effects S6 phosphorylation is a convergence point that incorporates multiple upstream signaling pathways in some tumor types. For example, in MAPKi sensitive melanoma, treatment with a RAFi or a MEKi, results in decreased pS6, whereas in the insensitive models no decrease in phosphorylation is observed [43]. The same may be true in the three PD-901 sensitive CRC cell lines (HCT116, LOVO, and WIDR) in contrast to the more resistant CRC cell line, GEO, which may indicate that decreased pS6RP may serve as a marker of responsiveness.

Next we transitioned our *in vitro* data *in vivo* using our CRC PDTX models. These models are thought to provide significant improvements over typical cell line xenografts, although clinical validation is ongoing [40], [44]. Similar to the *in vitro* data there were heterogeneous responses to combination treatment with one model, CUCRC040, demonstrating a statistically significant combination effect. This was in contrast to the CUCRC108 and CUCRC125 models, where the combination was only statistically different from the vehicle and to CUCRC042 which exhibited no treatment effects. Interestingly, this resistant PDTX model, exhibited an increase in pAKT when treated with the MEKi, PD-901, which was associated with an increase in pBAD, a protein that may have anti-apoptotic effects and lead to enhanced cell survival [45]. This may be worth pursuing as a putative resistance mechanism.

Despite strong preclinical evidence to support co-targeting of the PI3K/mTOR and MAPK pathways in several tumor types, early results of clinical trials have demonstrated mixed success [46]–[48]. The combinations were generally well tolerated and therapeutic doses were achieved with early evidence of anti-tumor activity observed in patients with advanced melanoma and low-grade serous ovarian cancer, whereas in patients with colorectal cancer tumor shrinkage was rarely documented.

In one Phase I study, colorectal cancer patients underwent prospective molecular profiling for mutations in KRAS, BRAF, PIK3CA and expression levels of PTEN and pMET. These patients were then offered first-in-human Phase I studies based on the results of these altered genetic/protein expression profiles. The choices included second-generation anti-EGFR mAbs (if KRAS wild-type), PI3K pathway inhibitors (if PIK3CA mutation or low PTEN expression), mTORC1 inhibitor plus anti-IGF1R mAb (if low PTEN expression), PI3K pathway inhibitors plus MEK inhibitors (if KRAS or BRAF mutation), BRAF inhibitor (if BRAF mutation) and anti-HGF mAb (if high pMET expression) [49]. Surprisingly, despite matching targeted agents based on molecular profiling, there was only 1 partial response observed in 68 patients. This patient had a PI3CA and KRAS mutation and was assigned to a PI3K inhibitor as monontherapy. Clearly the fact that these patients were heavily pre-treated and archival tissue was utilized for mutation characterization could have impacted these results, nonetheless, these results are provocative.

From this study and others, several principles have clearly emerged. First, the concept that targeting a mutation or pathway regardless of tissue of origin (the “one-size fits all” approach), has failed clinically. For example, targeting a BRAF mutation in melanoma is not the same as targeting the same mutation in colorectal cancer. This appears to be due to differential inherent and adaptive feedback/resistance mechanisms that exist among disease subtypes [50], [51]. Additionally, in colorectal cancer, matching patients with targeted therapy based on molecular profiling has thus far only been useful in eliminating, not selecting, therapy. The results we have presented here, in conjunction with other preclinical and clinical studies suggest that patient selection for combination therapy in CRC is complex and will likely rely upon factors beyond mutation detection. With newer technologies like next generation sequencing may lead to a more in depth molecular characterization (epigenetics, translocations, copy number variation analysis) of cancer cells. In addition, more extensive bioinformatics exploration can help guide better targeted combination therapies that can account for cancer with multiple drivers like colorectal cancer.

## Supporting Information

Figure S1 **Effect of single agent the PI3K/mTORi, PF-04691502, the MEKi, PD-0325901 or the combination in on PDTX models in athymic nude mice.** Growth curves of four patient derived tumor xeongraft models. Animals were treated daily for at least 28 days with vehicle, PF-502, PD-901 or the combination.
(TIFF)

Figure S2 **Effect of single agent the PI3K/mTORi, PF-04691502, the MEKi, PD-0325901 or the combination on downstream effector proteins assessed by antibody array.** Total protein was purified from PDTX at the end of treatment and assessed on an intracellular signaling antibody array.
(TIFF)
