# Supplementary figures and images for: Dual Pharmacological Targeting of the MAP Kinase and PI3K/mTOR Pathway in Preclinical Models of Colorectal Cancer
Source: PLoS One. 2014 Nov 17;9(11):e113037. doi: 10.1371/journal.pone.0113037 (PMC4234626; doi:10.1371/journal.pone.0113037)

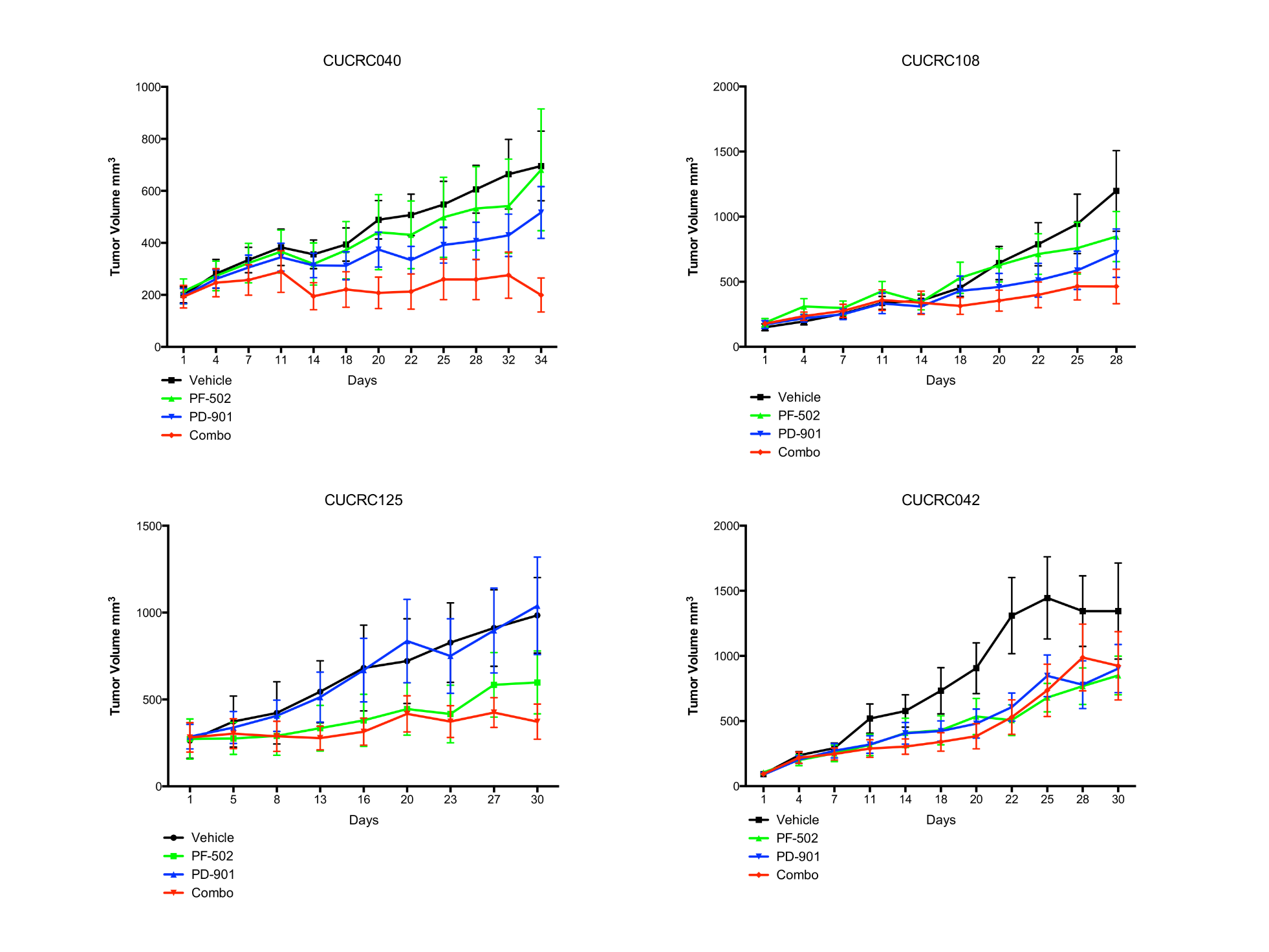

Supplement: Figure S1 — Effect of single agent the PI3K/mTORi, PF-04691502, the MEKi, PD-0325901 or the combination in on PDTX models in athymic nude mice. Growth curves of four patient derived tumor xeongraft models. Animals were treated daily for at least 28 days with vehicle, PF-502, PD-901 or the combination. (TIFF) [file pone.0113037.s001.tiff]

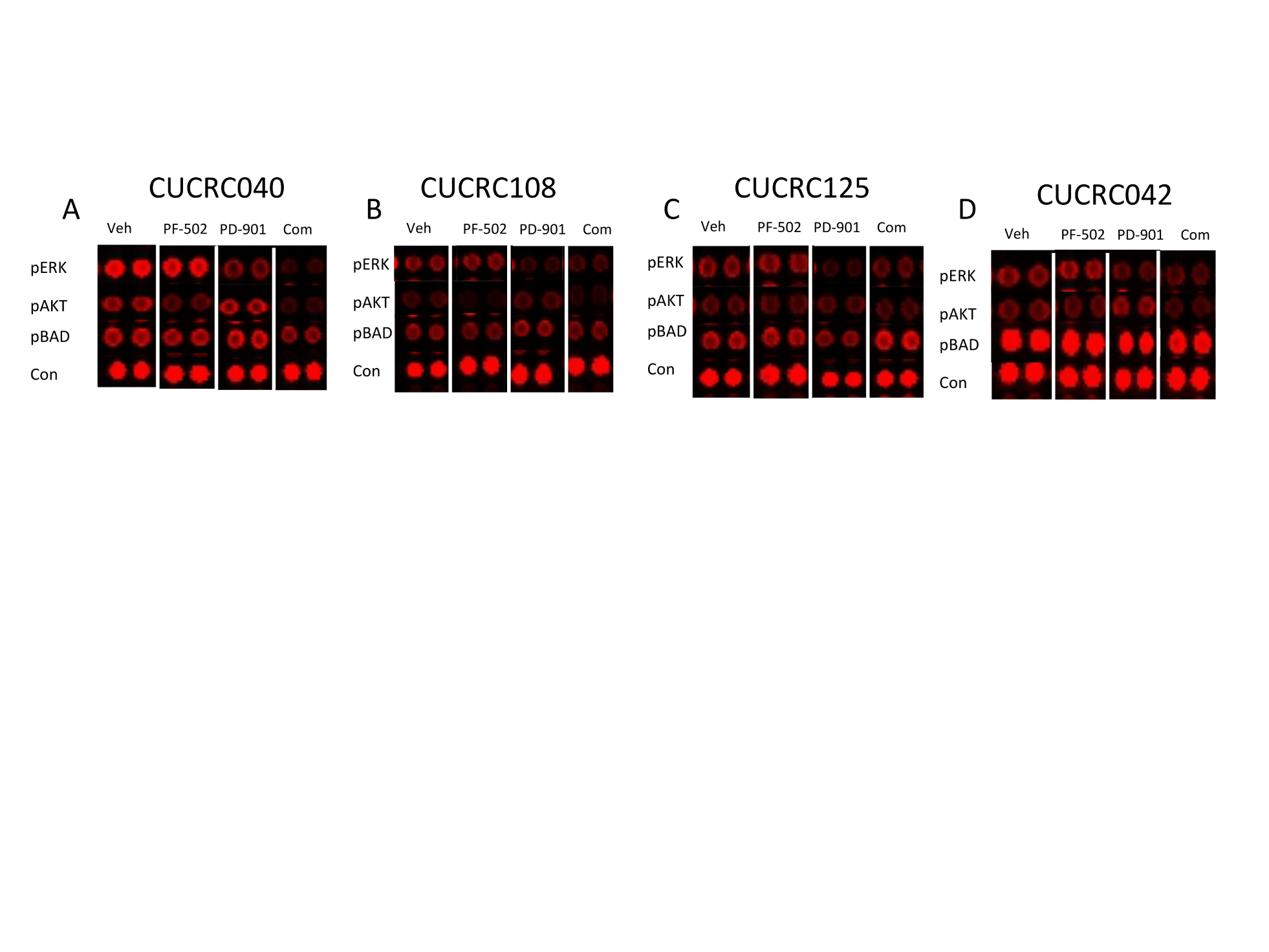

Supplement: Figure S2 — Effect of single agent the PI3K/mTORi, PF-04691502, the MEKi, PD-0325901 or the combination on downstream effector proteins assessed by antibody array. Total protein was purified from PDTX at the end of treatment and assessed on an intracellular signaling antibody array. (TIFF) [file pone.0113037.s002.tiff]
